# Allergen-Specific IgA Antibodies Block IgE-Mediated Activation of Mast Cells and Basophils

**DOI:** 10.3389/fimmu.2022.881655

**Published:** 2022-07-05

**Authors:** Yasmeen S. El Ansari, Cynthia Kanagaratham, Oliver T. Burton, Jenna V. Santos, Brianna-Marie A. Hollister, Owen L. Lewis, Harald Renz, Hans C. Oettgen

**Affiliations:** ^1^ Division of Immunology, Department of Pediatrics, Boston Children’s Hospital, Boston, MA, United States; ^2^ Institute of Laboratory Medicine, Philipps University Marburg, Marburg, Germany; ^3^ Department of Pediatrics, Harvard Medical School, Boston, MA, United States; ^4^ Laboratory of Lymphocyte Signaling and Development, The Babraham Institute, Cambridge, United Kingdom; ^5^ Institute of Laboratory Medicine, Universities of Giessen and Marburg Lung Center (UGMLC), Philipps University Marburg, German Center for Lung Research (DZL), Marburg, Germany

**Keywords:** basophils, basophil activation test, allergy, IgE, IgA, oral immunotherapy (OIT)

## Abstract

Mast cells and basophils have long been implicated in the pathogenesis of IgE-mediated hypersensitivity reactions. They express the high-affinity IgE receptor, FcϵRI, on their surface. Antigen-induced crosslinking of IgE antibodies bound to that receptor triggers a signaling cascade that results in activation, leading to the release of an array of preformed vasoactive mediators and rapidly synthesized lipids, as well as the *de novo* production of inflammatory cytokines. In addition to bearing activating receptors like FcεRI, these effector cells of allergy express inhibitory ones including FcγR2b, an IgG Fc receptor with a cytosolic inhibitory motif that activates protein tyrosine phosphatases that suppress IgE-mediated activation. We and others have shown that food allergen-specific IgG antibodies strongly induced during the course of oral immunotherapy (OIT), signal *via* FcγR2b to suppress IgE-mediated mast cell and basophil activation triggered by food allergen challenge. However, the potential inhibitory effects of IgA antibodies, which are also produced in response to OIT and are present at high levels at mucosal sites, including the intestine where food allergens are encountered, have not been well studied. Here we uncover an inhibitory function for IgA. We observe that IgA binds mouse bone marrow-derived mast cells (BMMCs) and peritoneal mast cells. Binding to BMMCs is dependent on calcium and sialic acid. We also found that IgA antibodies inhibit IgE-mediated mast cell degranulation in an allergen-specific fashion. Antigen-specific IgA inhibits IgE-mediated mast cell activation early in the signaling cascade, suppressing the phosphorylation of Syk, the proximal protein kinase mediating FcεRI signaling, and suppresses mast cell production of cytokines. Furthermore, using basophils from a peanut allergic donor we found that IgA binds to basophils and that activation by exposure to peanuts is effectively suppressed by IgA. We conclude that IgA serves as a regulator of mast cell and basophil degranulation, suggesting a physiologic role for IgA in the maintenance of immune homeostasis at mucosal sites.

## Introduction

IgE, while present at low concentrations in the serum, is unique in its ability to induce rapid and severe hypersensitivity responses. It is found tightly bound to basophils and mast cells *via* its high affinity-receptor, FcϵRI. Upon antigen binding, IgE-FcϵRI aggregation triggers a signaling cascade that results in the immediate release of preformed mediators and rapidly synthesized arachidonic acid metabolites followed by the production of pro-inflammatory cytokines. Mast cell mediators rapidly elicit a range of physiological responses including vasodilation and plasma extravasation, leading to tissue edema along with mucus production and smooth muscle constriction, all part of the immediate hypersensitivity response (1, 2). In addition to mediating these rapid physiologic changes, mast cells-via their delayed production of cytokines- serve a second but critical function in allergy as adjuvants for emerging type 2 adaptive immune responses. Mast cell-derived IL-4 drives IgE switching in B cells and promotes the expansion of pro-allergic lymphocytes, including type 2 innate lymphoid cells (ILC2) and Th2 cells, while suppressing the development and function of T regulatory cells (Treg) (3). Thus, the role of mast cells extends beyond driving immediate hypersensitivity reactions to include the induction and regulation of emerging immune responses (4, 5).

To date, a number of regulatory mechanisms that counter IgE-mediated activation of mast cells and basophils in immediate hypersensitivity responses have been described but their individual contributions to controlling allergic reactions in physiologic settings are unclear (6–8). IgE-mediated allergies are typically diagnosed based on skin prick testing or immunoassays. However, positive tests for IgE are not consistently predictive of clinical allergy. Despite being sensitized (e.g. detectable/measurable allergen specific IgE in the blood), some subjects with low to moderate levels of allergen-specific IgE will not experience any symptoms upon allergen exposure. Others will exhibit significant reactions. This is particularly evident in children undergoing testing for food allergies, creating clinical uncertainty that can only be resolved by performing food challenges to reliably establish allergy status (9–11). The absence of symptoms in some allergen IgE-sensitized children constitutes strong evidence for the presence and significant physiologic impact of factors that suppress IgE-mediated activation of mast cells and basophils.

Allergen-specific IgG antibodies have recently received attention as suppressors of allergic reactions to foods in subjects who harbor IgE. It is well established that allergen-specific IgG antibodies can inhibit IgE-mediated mast cell and basophil activation by two distinct mechanisms: 1) steric blockade of antigenic epitopes, or 2) signaling *via* the inhibitory Fc receptor FcγR2b (12, 13). Signals relayed *via* FcγR2b oppose activating input from FcϵRI, preventing downstream events that lead to mast cell and basophil degranulation (12, 14). In the setting of allergy, such IgG effects inhibit IgE-mediated immediate hypersensitivity reactions. The suppressive effects of IgG extend beyond immediate hypersensitivity. Studies using mouse models of food allergy have shown that allergen-specific IgG antibodies inhibit cytokine production by activated mast cells and basophils with immunomodulatory consequences for emerging adaptive immune responses including impaired Th2 expansion along with suppression and reprogramming of Tregs (15). Some compelling evidence regarding the relevance of inhibitory functions of IgG in allergy has come from studies showing that the appearance of allergen specific IgG coincides with natural resolution of milk and egg allergies in children (16, 17) and from parallel observations in respiratory allergy where the presence of IgG is inversely correlated with airway symptoms (18). Food-specific IgG antibody titers have recently been found to increase by several logs in patients who have completed oral immunotherapy (OIT). We and others have found that allergen/food specific IgG antibodies efficiently suppress the activation of IgE-sensitized basophils by allergen, which partly explains the food unresponsive state induced by OIT (14, 15, 19).

The most abundant antibody isotype in the intestine, where food allergens are encountered, is IgA. Thus, it would clearly be of interest to understand the effects of allergen specific IgA on IgE-mediated allergic reactions. IgA protects against mucosal pathogens through immune exclusion mechanisms by reducing the motility and adhesive capabilities of enteric pathogens. Strait and colleagues have suggested that IgA interactions might similarly impact the fate of ingested allergens, blocking their uptake and systemic absorption into the bloodstream. Like IgG, food-specific IgA antibodies increase dramatically after OIT (20). Some observations have pointed to correlations between impaired IgA responses and the development of allergic disease. Both high fecal and high salivary IgA have been correlated with a reduced risk of developing IgE associated diseases (21–23). A recent analysis of stool samples from healthy non-allergic individuals revealed a substantial amount of food allergen specific IgA is produced stably over time (24). Given its abundance and protective roles at mucosal surfaces, we hypothesized that IgA, like IgG, might inhibit IgE induced mast cell and basophil activation.

In this study we evaluated this hypothesis by testing the effects of antigen-specific IgA antibodies on IgE-mediated activation of murine mast cells and human basophils. We observed that IgA inhibits IgE-induced activation in an allergen-specific manner, and that IgA binding to murine mast cells was calcium- and sialic acid-dependent. 

## Materials and Methods

### Mouse Anti-TNP IgA Production and Purification

Frozen TIB-194 hybridoma cells (ATCC, Manassas, VA) were thawed according to manufacturer’s instructions, transferred and diluted with warmed RPMI-1640 (10% FBS, 100U/ml penicillin). Cells were centrifuged at 300 x g for 5 minutes, and the diluted freezing additive was removed and replaced with new media. Cells were propagated by the addition of fresh media approximately every 2-3 days. As cells multiplied, they were transferred into bigger flasks. Culture supernatants were tested by a sandwich ELISA for presence of anti-TNP IgA. Supernatants were collected and run over a protein L column (ThermoFisher Scientific, Waltham, MA). IgA was then concentrated and dialyzed with Amicon Ultra Centrifugal Filter Devices carrying a 100 kDa cut-off (Millipore Sigma, Darmstadt, Germany) and filter-sterilized with 0.2μM syringe filters (Millex, EMD Millipore, Billerica, MA).

### Mast Cell Culture

BALB/cJ mouse bone marrow was cultured in RPMI-1640 media supplemented with 10% fetal bovine serum (Atlanta Biologicals, Lawrenceville, GA), 10mM HEPES buffer, 100 μg/ml streptomycin, 100U/ml penicillin 10μg/ml gentamicin, 1% Minimum Essential Medium non-essential amino acids, 1 mM sodium pyruvate, and 55μm 2-mercaptoethanol, (all from ThermoFisher Scientific, Waltham, MA) in the presence of both 10-20 ng/ml of IL-3 and SCF (Shenandoah Biotechnology, Warwick, PA) for the differentiation into BMMCs. BMMCs were cultured for 4-6 weeks until cultures reached 90% purity. The purity of BMMCs were assessed by flow cytometry (c-Kit^+^ FcϵRIα^+^).

For the lysosomal-associated membrane protein 1 (LAMP-1) assay, mouse BMMCs were incubated overnight with anti-TNP IgE (50 ng/ml, BD Biosciences, Franklin Lakes, NJ), and anti-TNP IgA (100 μg/ml, TIB-194 hybridoma, ATCC, Manassas, VA). Cells are then washed with RPMI to remove unbound antibody, and then stimulated for 10 mins at 37°C with TNP BSA (50 ng/ml, Biosearch Technologies, Teddington, UK) while simultaneously being stained with, BV605 anti-c-Kit (Biolegend, San Diego,CA), fixable viability dye eFluor 780 (eBioscience, San Diego, CA), and PE anti-LAMP-1. For antigen specificity experiments, anti-OVA IgA (gifted by Dr. Duane Wesemann), was used alongside anti-TNP IgA.

For imaging experiments, BMMCs were IgE/IgA sensitized as per the conditions above, and stained with NucBlue Live Cell Stain (Thermofisher Scientific, Carlsbad, CA), and anti-mouse Fc block and FITC anti-IgA (BD Biosciences, Franklin Lakes, NJ), or isotype control. Cells were then cytospun onto a slide. Slides were visualized and imaged using an EVOS M7000 microscope (Thermofisher Scientific, Bothell, WA).

For measurements of phospho-Syk, mouse BMMCs were incubated with anti-TNP IgE, and anti-TNP IgA as above, and stimulated at 37°C for zero, one and two minutes. Cells were fixed and stained using Thermofisher Scientific Protocol C: Two-step Protocol for Fixation/Methanol. Cells were stained with mouse PE anti-phospho-Syk (Cell Signaling Technologies, Danvers, MA), in the presence of mouse Fc block and assessed on an LSR Fortessa.

For phospho-Syk/Syk western blots, 10^6^ BMMCs were sensitized overnight with anti-TNP IgE, and anti-TNP IgA as described in conditions above. After sensitization, cells were stimulated with 100 ng/mL of TNP-OVA for 0–3 minutes. Cold FACS buffer was used to stop stimulation, and cells were pelleted and lysed with radioimmunoprecipitation assay buffer (Sigma-Aldrich) containing a cocktail of protease and phosphatase inhibitors (MilliporeSigma, Burlington, Mass).Lysed cells were then spun down to collect protein supernatant. Twenty microliters of lysate were resolved on a 4-15% MiniProtean TGX precast gel (Biorad, Hercules, Calif) and electrophoretically transferred to a polyvinylidene fluoride (PVDF) membrane (MilliporeSigma). Membranes were blocked with 5% BSA in tris-buffered saline with 0.05% tween 20 (TBST) for 1 hour. Membranes were probed with primary antibodies overnight, washed with TBST, and then probed with appropriate peroxidase-conjugated secondary antibodies for 1 hour. Membranes were developed with Supersignal chemiluminescent substrate reagent and imaged on an iBright Western Blot scanner (Thermo Fisher Scientific, Waltham, Mass).

### Cytokine Release Assay

For the cytokine release assay, BMMCs were stimulated for 6 hours. Supernatants were collected and cytokines were measured using the Cytometric Bead Array (CBA) Mouse/Rat Soluble Protein Master Buffer kit, along with the cytokine flex sets for IL-13, IL-6, and TNF-α according to manufacturer’s instructions (BD Biosciences, Franklin Lakes, NJ).

### Flow Cytometry and Immunofluorescence IgA Binding Studies on BMMCs, Splenic T Cells and Peritoneal Cells

T cells were purified from a BALB/cJ spleen by MACS sorting using the CD3ε Microbead kit according to manufacturers instructions (Miltenyi Biotec, Bergisch Gladbach, Germany). BMMCs were incubated with 100 μg/ml anti-TNP IgA or anti-TNP OVA in 1X HBSS for 30 minutes at 4°C. Purified T cells were also incubated with 100 μg/ml anti-TNP IgA in 1X HBSS for 30 minutes at 4°C. Mast cells were stained for BV605 anti-c-Kit, PE anti-FcϵRI, fixable viability dye eFluor 780 (eBioscience, San Diego, CA), and FITC anti-IgA (BD Biosciences, Franklin Lakes, NJ) in the presence of anti-mouse Fc block (Biolegend, San Diego, CA), in FACS buffer (1X PBS, 10% FBS) at 4°C for 30 minutes. T cells were stained for PE anti-CD3 (eBioscience, San Diego, CA), BV605 anti-CD4 (Biolegend, San Diego, CA), FITC anti-IgA (BD Biosciences, Franklin Lakes, NJ), and fixable viability dye eFluor 780 (eBioscience, San Diego, CA), in FACS buffer.

Peritoneal lavage was isolated from BALB/cJ by injection of 5 ml of 1X HBSS into the peritoneal cavity to dislodge attached cells. Cells were pelleted and resuspended in 1X HBSS containing 100 μg/ml of anti-TNP IgA – incubated for 30 minutes at 4°C. Cells were then washed in FACS buffer and stained with mouse AF700 anti-CD45 (Biolegend, San Diego, CA), APC anti-CD11b (eBioscience, San Diego, CA), BV605 anti-c-Kit (Biolegend, San Diego, CA), PE anti-FcϵRIα (Biolegend, San Diego, CA), and FITC anti-IgA (BD Biosciences, Franklin Lakes, NJ.

### Deglycosylation and Desialylation of IgA

For removal of N-linked glycans, anti-TNP IgA was incubated with PNGase F (New England Bioscience, Ipswich, MA) at 37°C for 72 hours according to manufacturer’s instructions. For desialylation, anti-TNP IgA was digested with neuraminidase from *Vibrio cholerae* (Sigma, Saint Louis, MO) at 37°C for 24 hours according to manufacturer’s instructions. The reactions (control IgA in glycobuffer, and digested IgA in glycobuffer and neuraminidase) were then incubated at 65°C for 10 min. to deactivate the enzymes. Digestion efficacy was assessed by lectin blot analysis.

### Human IgA Purification and Anti-Peanut IgA Quantification From Post OIT Sera

As OIT induces a strong food allergen-specific IgA response, we used sera obtained from patients who had undergone peanut OIT as a source of peanut-specific IgA (25). Post OIT sera were pooled from several patients and IgA was purified using an anti-human IgA CaptureSelect affinity column as per manufacturer’s instructions (ThermoFisher Scientific, Carlsbad, CA). The IgA-enriched eluate from this column was then run through a Protein G column as per manufacturer’s instructions to further deplete it of IgG (ThermoFisher Scientific, Carlsbad, CA). The IgA was concentrated to a volume equivalent to that of the original input serum and dialyzed with Amicon Ultra Centrifugal Filter Devices carrying a 100 kDa cut-off (Millipore Sigma, Darmstadt, Germany) and filter-sterilized with 0.2 μm syringe filters (Millex, EMD Millipore, Billerica, MA). Total IgA and IgG was quantified using the Human IgA and IgG ELISA kits (ThermoFisher Scientific, Carlsbad, CA).

### Human Basophil Activation Test

Basophil activation tests (BAT) were performed using the Flow CAST Basophil Activation Test kit (Bühlmann Laboratories, Schönenbuch, Switzerland). 50 μl aliquots of whole blood from peanut allergic patients were pre incubated with 125-1000 μg/ml of purified IgA for 2-4 hours at 37°C in 100 μl of basophil stimulation buffer. Samples were incubated for l5 min at 37°C with 3.6x10^-4^ μg/ml of crude peanut extract (CPE) and staining antibodies for human PE anti-CCR3 (Biolegend, San Diego, CA) and FITC anti-CD63 (BD Biosciences, Franklin Lakes, NJ). Anti-FcϵRI stimulation was used as a positive control for basophil activation. After red blood cell lysis, cells were washed and assessed for activation by flow cytometry. Basophils were identified as SSC^low^CCR3^+^ and activated cells identified based on CD63 expression. Around 200 basophils were evaluated for each sample. Peripheral blood from peanut allergic donors was obtained with informed consent under a protocol approved by the Institutional Review Board of Boston Children’s Hospital.

### Fluorescent Labeling of Human IgA and IgA Binding Studies on Human Cell Types

Human IgA purified from post OIT sera was fluorescently labeled using the Pierce FITC antibody labeling kit (Thermofisher Scientific, Rockford, IL) according to the manufacturer’s instructions. For IgA binding experiments, donor whole blood was stained with labeled IgA, along with antibodies to human leukocyte markers PE anti-CCR3, APC anti-FcεR1, AF700 anti-CD3, PE-Cy7 anti-CD16, BV510 anti-CD14 all from (Biolegend, San Diego, CA) for 30 minutes at 37°C.

## Results

### IgA Antibodies Inhibit IgE-Induced Mouse Mast Cell Degranulation in an Antigen-Specific Manner

In order to evaluate the effects of IgA antibodies on IgE-mediated mast cell activation, we took advantage of the availability of monoclonal hapten-specific IgA antibodies as well as the well-characterized experimental model system of cultured BMMCs in which murine bone marrow cells are incubated with IL-3 and SCF to differentiate to c-Kit^+^ FcϵRI^+^ mast cells. Our own investigations as well as others have shown that IgG antibodies can inhibit IgE-mediated BMMC activation in a manner that is antigen specific and FcγR2b -dependent (e.g. the effect is not observed in FcγR2b^-/-^ BMMCs) (14, 15). To test the effects of IgA on IgE-mediated mast cell activation, BMMCs were incubated with IgE ± IgA monoclonal antibodies specific for the hapten, trinitrophenyl (TNP) prior to antigen exposure. Cells were then washed to remove unbound antibody, and then stimulated for 10-minutes with (TNP-BSA). LAMP-1, which is extruded to the plasma membrane surface during granule fusion, was measured by flow cytometry as an indicator of mast cell degranulation.

**Figure 1:**
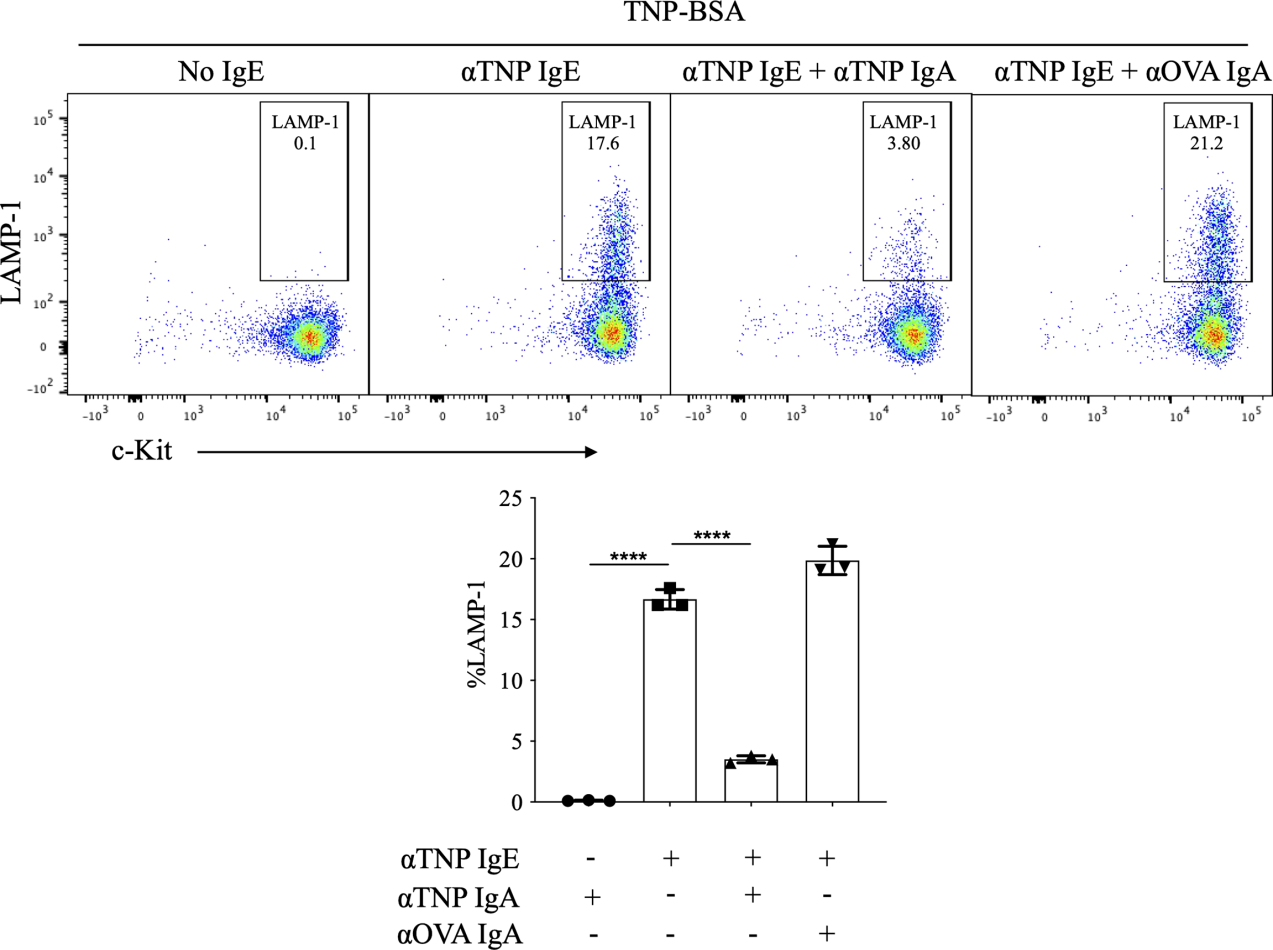
Effects of IgA antibodies on IgE mediated degranulation of bone marrow derived mast cells. Flow cytometry plots from a representative experiment (left) and aggregate data (n=3) bar plots (right) of percent LAMP-1 expression of IgE sensitized BMMCs following antigen exposure. BMMCs were sensitized with anti-TNP IgE (αTNP IgE) (50 ng/ml). Subsequently, some cells were co-incubated with anti-TNP IgA (αTNP IgA) (100 µg/ml) (purified from TIB-194 hybridoma) or anti-OVA IgA [αOVA IgA (100 µg/ml)]. Cells were primed with antibodies overnight, then washed and stimulated with, 50 ng/ml of TNP-BSA for 10 minutes before assessing activation by staining with anti-mouse c-Kit, and LAMP-1. Statistical analysis done by ANOVA. Data shown mean ± SEM of one experiment representative of three independent experiments. ****P < .0001.

In the absence of anti-TNP IgE, TNP-BSA induced no detectable BMMC degranulation, with LAMP-1 expression only 0.1% ± 0.02% (**Figure 1**). Anti-TNP IgE-sensitized BMMCs exposed to TNP-BSA exhibited a robust induction of surface LAMP-1 on the cell surface (16.9% ± 0.5%). Addition of anti-TNP IgA to these cultures resulted in a marked suppression of IgE-induced LAMP-1 expression (3.5% ± 0.2%) (**Figure 1**). In contrast, incubating anti-TNP IgE sensitized mast cells with IgA directed against an irrelevant antigen, ovalbumin (OVA), did not impair IgE mediated activation (20.5% ± 0.7%), indicating that IgA mediated suppression is antigen specific. While anti-OVA IgA did not suppress TNP-IgE responses, we confirmed that it did still bind to BMMCs in a similar manner to anti-TNP IgA (**Figure S1**).

### IgA Binds to Bone Marrow Derived Mouse Mast Cells and Peritoneal Mouse Mast Cells

**Figure 2:**
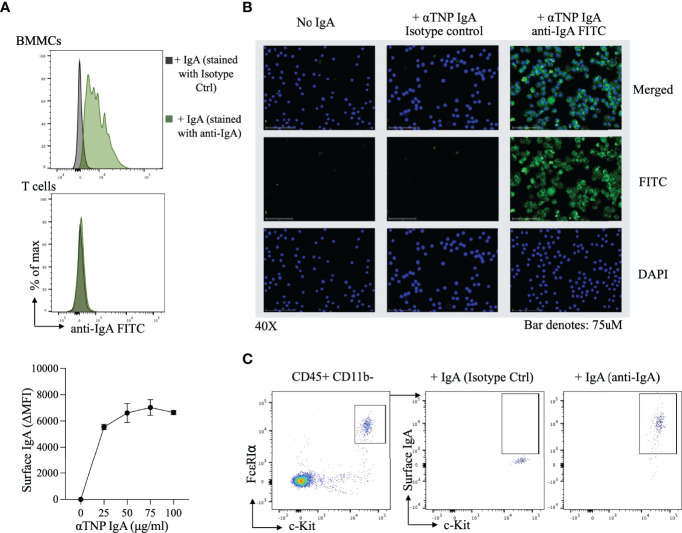
Surface IgA staining on bone marrow derived mast cells and peritoneal mast cells **(A)** *Flow-cytometric evaluation of IgA binding to BMMCs.* Representative histogram of αTNP IgA binding to BMMCs (top) and purified splenic T cells (middle), and a dose response curve of αTNP IgA binding to BMMCs. Mast cells incubated with or without anti-TNP IgA were stained with antibodies for c-Kit, FcϵRIα, and IgA and analyzed by flow cytometry. Purified splenic T cells incubated with or without anti-TNP IgA were stained with antibodies for CD3, CD4, and IgA and analyzed by flow cytometry. Data shown is one experiment representative of six independent experiments. **(B)** *Immunofluorescence analysis of IgA binding to BMMCs.* Representative photomicrographs (40x) of BMMCs incubated with or without 100µg/ml of αTNP IgA in HBSS for 30 minutes at 4°C. BMMCs were stained for nucleus (blue), IgA (green), or isotype control (middle), and merged (right). Scale bars: 75 µm. Images were taken using an EVOS M7000 microscope. Data shown is one experiment representative of two independent experiments. **(C)** *Flow-cytometric evaluation of IgA binding to peritoneal mast cells.* Representative flow cytometry plots of IgA staining on peritoneal cavity mast cells, identified by the presence of CD45, c-Kit, FcϵRIα. Data shown is one experiment representative of two independent experiments.

The observation that IgA antibodies blocked IgE-induced activation of allergen-exposed BMMCs, led us to consider the possibility that IgA might be physically interacting with these mast cells. Surface expression of IgA on c-Kit^+^ FcϵRI^+^ cells was measured by flow cytometry following incubation of BMMCs with anti-TNP IgA for 30 minutes in HBSS. In the presence of IgA, BMMCs uniformly stained with anti-IgA FITC in a dose dependent manner (**Figure 2A**, upper and lower panel), however no IgA binding was observed with purified splenic T cells. Immunofluorescence imaging of BMMCs, incubated with or without anti-TNP IgA similarly revealed the presence of staining (green) on mast cells incubated with IgA (**Figure 2B**). In order to evaluate IgA binding to primary mast cells, we examined peritoneal lavage cells. Staining for surface IgA after incubation with anti-TNP IgA, revealed IgA binding to CD11b^-^ c-Kit^+^ FcϵRI^+^ mast cells isolated from the peritoneum (**Figure 2C**).

### IgA Binding to Mast Cells Is Calcium- and Sialic Acid-Dependent

**Figure 3:**
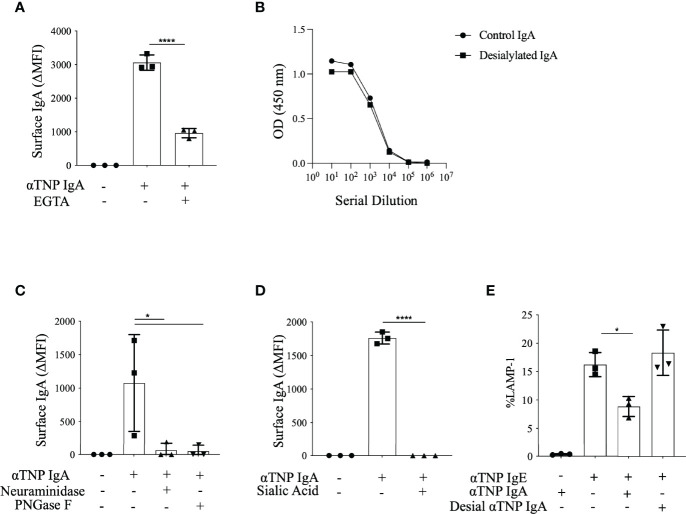
Effects of calcium and sialic acid on IgA binding to BMMCs. **(A)** *Effect of calcium chelation on IgA binding.* Mean fluorescence intensity (MFI) of BMMCs stained with anti-IgA after incubation with anti-TNP IgA in the absence or presence of EGTA. **(B)** *Retention of TNP-OVA binding by desialylated IgA.* Serial dilutions of desialylated or buffer control treated anti-TNP IgA were tested for TNP-OVA binding by ELISA. **(C)** *Analysis of sialic acid requirement for IgA binding*. MFI of BMMCs incubated with untreated anti-TNP IgA, desialylated anti-TNP IgA or N-deglycosylated anti-TNP IgA in HBSS for 30 minutes at 4°C. Mast cells incubated with or without anti-TNP IgA were stained with antibodies for c-Kit, FcϵRIα, and IgA and analyzed by flow cytometry. **(D)** *Effects of sialic acid competition on IgA binding to BMMCs.* MFI of BMMCs stained with anti-IgA with or without preincubation with sialic acid **(E)** *Consequences of sialic acid removal on the inhibitory effects of IgA*. Bar plot of percent degranulation across three replicates of LAMP-1 induction in IgE sensitized BMMCs incubated with or without anti-TNP IgA, or desialylated anti-TNP (desial αTNP) IgA as in Figure 3A. Statistical analysis done by one-way analysis of variance (ANOVA). Data shown mean ± SEM of one experiment representative of three independent experiments. *P < .05, and ****P < .0001.

As mast cells do not express any known IgA receptors, we sought to characterize the physical properties of IgA binding to BMMCs. To test calcium dependence, exogenous calcium was chelated by addition of EGTA to BMMC suspensions prior to incubation with IgA. Calcium removal dramatically impaired IgA binding with a decrease in MFI from 3060 ± 13.4 to 961 ± 79.8 (**Figure 3A**), but not IgE binding (**Figure S2**). We similarly observed that BMMCs suspended in calcium-free PBS failed to bind IgA (data not shown).

The abrogated IgA binding after calcium chelation suggested interaction with a calcium dependent receptor. Some such receptors are lectins, leading us to consider that the carbohydrate moieties on IgA might be mediating its interaction with BMMCs. To test this, we treated anti-TNP IgA with either PNGase F to remove all N-linked sugars, or neuraminidase to remove terminal sialic acid residues. Either N-deglycosylation of anti-TNP IgA or the removal of sialic acid rendered the antibody completely unable to bind to BMMCs (**Figure 3C**) without affecting its ability to bind to TNP-OVA (**Figure 3B**). To corroborate this sialic acid dependence of IgA binding to BMMCs, we incubated cells with sialic acid for 30 minutes prior to addition of IgA and observed suppression of IgA binding to undetectable levels (**Figure 3D**). The suppression of this response is dose dependent (data not shown). Using desialylated IgA, we also confirmed a functional requirement for sialic acid in the ability of IgA to suppress IgE-mediated mast cell activation. We observed that while untreated IgA inhibited LAMP-1 induction (of 8.8% ± 0.06% in treated compared to 16.2% ± 1.2% in controls), desialylated anti-TNP IgA was incapable of suppressing IgE-mediated BMMC degranulation (18.3% ± 2.3%) (**Figure 3E**). We could not similarly establish the effects of calcium depletion on the inhibitory function of IgA antibodies since it is not possible to perform mast cell activation assays in the absence of calcium. These experiments reveal that the binding of IgA to BMMCs is dependent both on ambient calcium concentration and on sialylation of the IgA antibodies and that sialyation is also critical for functional inhibition of mast cell activation by IgA.

### IgA Inhibits Syk Phosphorylation and Cytokine Production in IgE-Activated Bone Marrow Derived Mast Cells

**Figure 4:**
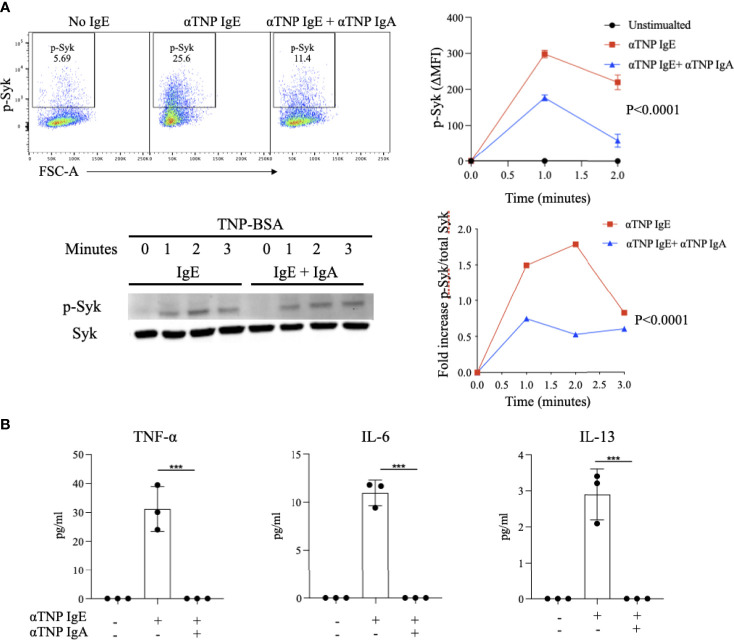
Effect of IgA antibodies on IgE-induced phosphorylation of Syk and on cytokine production by activated bone marrow derived mast cells **(A)** *IgA effects on Syk phosphorylation*. IgE sensitized BMMCs were incubated with or without anti-TNP IgA and stimulated with antigen for up to two minutes followed by measurement of phosphorylated-Syk (phospho-Syk) using flow cytometry (upper panel). Representative Syk phosphorylation blots and compiled ratios of phospho protein/total protein intensities in anti-TNP IgE-sensitized BMMCs or anti-TNP IgE/IgA treated BMMCs (lower panel). This experiment is representative of three replicates. **(B)** *IgA effects on cytokine production*. Cytokine (IL-6, IL-13 and TNF-α) levels in the supernatants of anti-TNP IgE sensitized BMMC incubated with or without anti-TNP IgA and stimulated with TNP-BSA for 6 hours. Statistical analysis done by ANOVA. Data shown mean ± SEM of one experiment representative of three independent experiments. ***P < .001.

IgE-antigen receptor crosslinking on BMMCs results in the phosphorylation of the protein tyrosine kinase (PTK) Syk. Activation of a PTK signaling cascade downstream of Syk drives many of the phenotypes of activated mast cells, including degranulation, synthesis of arachidonate-derived lipid mediators and induction of cytokine transcription. In order to establish if IgA antibodies block this critical signaling pathway, we evaluated their effects on phosphorylated-Syk (p-Syk), the active signaling form of this PTK. BMMCs loaded with anti-TNP IgE and stimulated with TNP-BSA exhibited rapid increases in p-Syk levels, peaking one minute after stimulation (MFI 298 ± 7.6) and then quickly decreasing (**Figure 4A**). In contrast, allergen-exposed BMMCs sensitized with anti-TNP IgE and also incubated with anti-TNP IgA exhibited attenuated p-Syk induction (176.6 ± 4.7) 1 minute after stimulation. To further validate these results as well as to normalize p-Syk to total Syk, we performed phospho-western blot analysis. This similarly showed Syk phosphorylation detectable as early as 1 minute after stimulation (**Figure 4A**). Quantification of the ratio of signal intensities of phospho-Syk to total Syk revealed that phosphorylation induced by IgE was less at all time points in BMMCs that were also incubated with IgA.

Cytokine production is an important property of activated mast cells and is critical for the generation and tissue recruitment of the effector cells of inflammation as well as for the expansion of type 2 adaptive immune responses. We evaluated the effects of antigen-specific IgA on IgE-induced cytokine production by BMMCs. As expected, IgE-sensitized BMMCs exposed to antigen produce IL-13, TNF-α, and IL-6. Addition of IgA to these IgE-sensitized BMMCs prior to antigen stimulation resulted in a complete suppression of the production of these cytokines (**Figure 4B**). These results indicate that IgA acts early in the FcεRI signaling cascade, inhibiting formation of the most proximal signaling intermediate, p-Syk and that IgA-mediated blockade of mast cell activation extends beyond degranulation to also affect cytokine production.

### IgA Antibodies Inhibit the Peanut-Induced Activation of Basophils From Allergic Subjects

**Figure 5:**
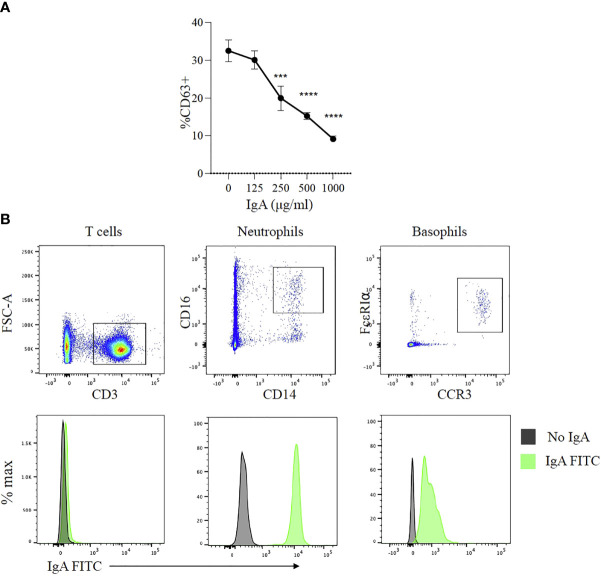
Effects of peanut-specific IgA on basophils from peanut allergic subjects **(A)** *IgA effects on allergen induced degranulation.* Dose response analysis of effects of IgA from peanut allergic subjects on peanut induced activation of basophils from a peanut allergic donor. Data shown from one experiment run in triplicate. Statistical analysis by ANOVA. ***P < .001, ****P < .0001 **(B)** *IgA binding to basophils*. Representative flow cytometry plots showing human IgA purified from post-OIT sera labeled with FITC binding to T cells, neutrophils and basophils from whole blood. T cells were identified by the presence of CD3, neutrophils by CD45, CD14, CD16, and basophils by CCR3 and FcεRI.

Peanut-specific IgG and IgA antibodies are both present in the plasma of subjects with peanut allergy and both of their levels increase after OIT. We have observed that IgE-mediated activation of basophils is potently suppressed by IgG signaling *via* FcγR2b (14). We took advantage of post OIT sera as a source of PN specific IgA to test whether IgA exerts similar protective effects as PN-IgG in peanut allergic patients. IgA was enriched from these patient sera by affinity chromatography and further purified by IgG depletion using Protein G Sepharose, yielding a preparation containing 2.5 mg/ml IgA and no detectable IgG. Whole blood samples from a peanut allergic donor (with confirmed IgE mediated allergy to peanut) were pre-incubated for two to four hours with varying concentrations of purified IgA (125-1000 μg/ml). The samples were then stimulated with CPE and expression of CD63 was used to as a marker of basophil activation. Basophil activation was markedly suppressed in the presence of IgA (**Figure 5A**). This inhibitory effect of IgA was dose dependent over a range of IgA amounts from(250-1000 μg/ml) (**Figure 5A**). While IgA has been found to interact with several human cell types, including neutrophils and eosinophils (26, 27), its interaction with mast cells and basophils is incompletely characterized. At least one report has suggested an activating effect of IgA antibodies on basophils (28). We sought to determine if IgA might bind to basophils. We stained whole blood with fluorescently labeled human IgA along with antibodies defining the key leukocyte subsets. As expected, IgA was bound by neutrophils but not by T cells (**Figure 5B**). Basophils also exhibited IgA binding with a unimodal fluorescence shift relative to isotype control. These observations indicate that IgA antibodies, like IgG antibodies, bind to and are capable of inhibiting the activation of antigen stimulated basophils from peanut-allergic subjects.

## Discussion

As the most abundant immunoglobulins at mucosal surfaces, IgA antibodies are well-positioned to act as the initial humoral immune sentinels for ingested and inhaled antigens. They have been extensively characterized with respect to their roles in defense in the airway and enteric infection, maintenance of gut microbial homeostasis, and regulation of immune responses (29). Depending on the targeted effector cell and disease model being studied, both pro- and anti-inflammatory properties have been attributed to IgA (26). The physiological functions of IgA in regulating mast cell biology in allergic disease have not been extensively studied.

We took advantage of an array of experimental tools including murine cultured mast cells, primary peritoneal mast cells, human basophils, monoclonal hapten-specific IgA, and polyclonal IgA purified from the sera of peanut allergic subjects to test the hypothesis that antigen-specific IgA antibodies inhibit FcϵRI mediated mast cell and basophil activation. We found that IgA binds to mast cells in a manner dependent on calcium and sialic acid and inhibits IgE-induced mast cell degranulation. IgA exerts its function at a receptor-proximal point in the FcεRI-initiated PTK signaling cascade by decreasing phosphorylation of Syk. In addition to blocking degranulation and PTK signaling pathways, we found that IgA suppresses IgE-induced, cytokine production by mast cells *in vivo*. The relevance of these findings to humans was demonstrated in our confirmation that IgA has suppressive effects on the allergen-induced activation of basophils from peanut allergic subjects.

Our findings provide new insights into regulatory roles IgA might play in allergic disease, most notably its ability to suppress IgE-induced mast cell activation. Strait and colleagues have previously reported that IgA antibodies can suppress IgE-mediated anaphylaxis in mice (20). They showed that the suppressive effect of IgA is observed even in knockout mice lacking the murine IgA receptor, Fcα/μR and conclude that IgA likely confers protection by steric blockade of TNP epitopes on absorbed food allergens rather than by a receptor-mediated inhibitory mechanism. In contrast, some of the observations in our study suggest that IgA mediated inhibition of mast cell activation may in fact be receptor mediated. Specifically, we demonstrate IgA binding to murine mast cells and neutrophils but not lymphocytes, indicating a cellular restriction of IgA interaction suggestive of receptor-mediated binding. We further observed that IgA association with mast cells is calcium-dependent and that both binding and suppressive function of IgA require the presence of sialic acid. These properties are consistent with a receptor-mediated interaction leading us to consider the possibility that a member of the C-type lectin family of receptors might be exerting the inhibitory effect of IgA. However, in our screening experiments using BMMCs from mice with targeted deletions of some of the members of this family known to be expressed on mast cells (including SIGN-R1, CD33, and Siglec F) IgA mediated inhibition of IgE activation remained intact (data not shown). Based on reports that IgA might signal *via* FcγR2b, with Galectin-3 as a binding intermediate (30), we additionally tested IgA’s suppressive effects on BMMCs from mice with a targeted deletion of FcγR2b, and found that suppression remained intact (data not shown). Thus, while our results provide some evidence for a receptor-mediated inhibitory effect of antigen-specific IgA on mast cells, further studies will be required to identify the relevant receptor mediating this effect.

Our finding that IgA contained in the serum of subjects with food allergy can suppress peanut-induced activation of basophils from an allergic subject, points to a potential regulatory function of IgA. A role of IgA in food tolerance has previously been suggested by a number of clinical observations. Young children with allergies to cow’s milk typically outgrow these allergies later in childhood. The appearance of IgA antibodies specific for cow’s milk has been noted to trend in parallel with this resolution of allergy (31). Active induction of food unresponsiveness by OIT is also correlated with the induction of IgA responses. We initially found marked increases in allergen-specific IgA in a trial of OIT for peanut an observation that has subsequently been extended by others to cow’s milk and egg OIT (14, 32–35). Murine models have also documented specific IgA responses following food allergen ingestion and OIT (24, 36–38). Despite the consistent observation of association between IgA responses and food tolerance in humans and in murine models of food allergy, neither the direct contributions of IgA to tolerance nor the mechanisms thereof have been established. Immune exclusion, in which IgA antibodies prevent food allergen uptake by intestinal M cells is one proposed mechanism (39). We believe that the findings presented in our current study offer another likely alternative whereby IgA antibodies suppress allergic reactions in a receptor-mediated manner at the effector cell level.

Defining the mechanisms contributing to tolerance of ingested antigens is of critical importance in the field of food allergy. This report provides important new insights in this regard, demonstrating that IgA antibodies, which are known to be physiologically induced after food ingestion and OIT, can block IgE-mediated activation of mast cells and basophils and consequent hypersensitivity reactions. It will be of great interest, going forward to further delineate the mechanisms underlying this effect, and to consider whether the induction of IgA responses or passive administration of allergen-specific IgA might be utilized as therapies.

## Data Availability Statement

The original contributions presented in the study are included in the article/**Supplementary Material**. Further inquiries can be directed to the corresponding author.

## Ethics Statement

The studies involving human participants were reviewed and approved by The Institutional Review Board at Boston Children’s Hospital. Written informed consent to participate in this study was provided by the participants’ legal guardian/next of kin. The animal study was reviewed and approved by Institutional Animal Care and Use Committee and Animal Research Children’s Hospital.

## Author Contributions

YSE, HR, and HCO designed experiments, interpreted the results, and wrote the paper. Experiments were performed by YSE, OTB, CK and OLL. JS and B-MH recruited peanut allergic patients for the basophil activation tests. All authors contributed to the article and approved the submitted version.

## Funding

HCO was supported by NIH grant 2R01AI119918-06 and the Food Allergy Science Initiative.

## Conflict of Interest

The authors declare that the research was conducted in the absence of any commercial or financial relationships that could be construed as a potential conflict of interest.

## Publisher’s Note

All claims expressed in this article are solely those of the authors and do not necessarily represent those of their affiliated organizations, or those of the publisher, the editors and the reviewers. Any product that may be evaluated in this article, or claim that may be made by its manufacturer, is not guaranteed or endorsed by the publisher.

## Supplementary Material

Supplementary Figure 1 Surface anti-OVA IgA staining on bone marrow derived mast cells. Representative histogram of αTNP IgA, and αOVA IgA binding to bone marrow derived mast cells (BMMCs). Mast cells incubated with or without anti-TNP IgA or anti-OVA IgA were stained with antibodies for c-Kit, FcϵRIα, and IgA and analyzed by flow cytometry. Data shown representative of one experiment run twice independently.

Supplementary Figure 2 Effects of calcium on IgE binding to BMMCs. Mean fluorescence intensity (MFI) of BMMCs stained with anti-IgE after incubation with anti-TNP IgE in the absence or presence of EGTA. Data shown representative of one experiment run twice independently.
