# Supplementary figures and images for: Allergen-Specific IgA Antibodies Block IgE-Mediated Activation of Mast Cells and Basophils
Source: Front Immunol. 2022 Jul 5;13:881655. doi: 10.3389/fimmu.2022.881655 (PMC9294179; doi:10.3389/fimmu.2022.881655)

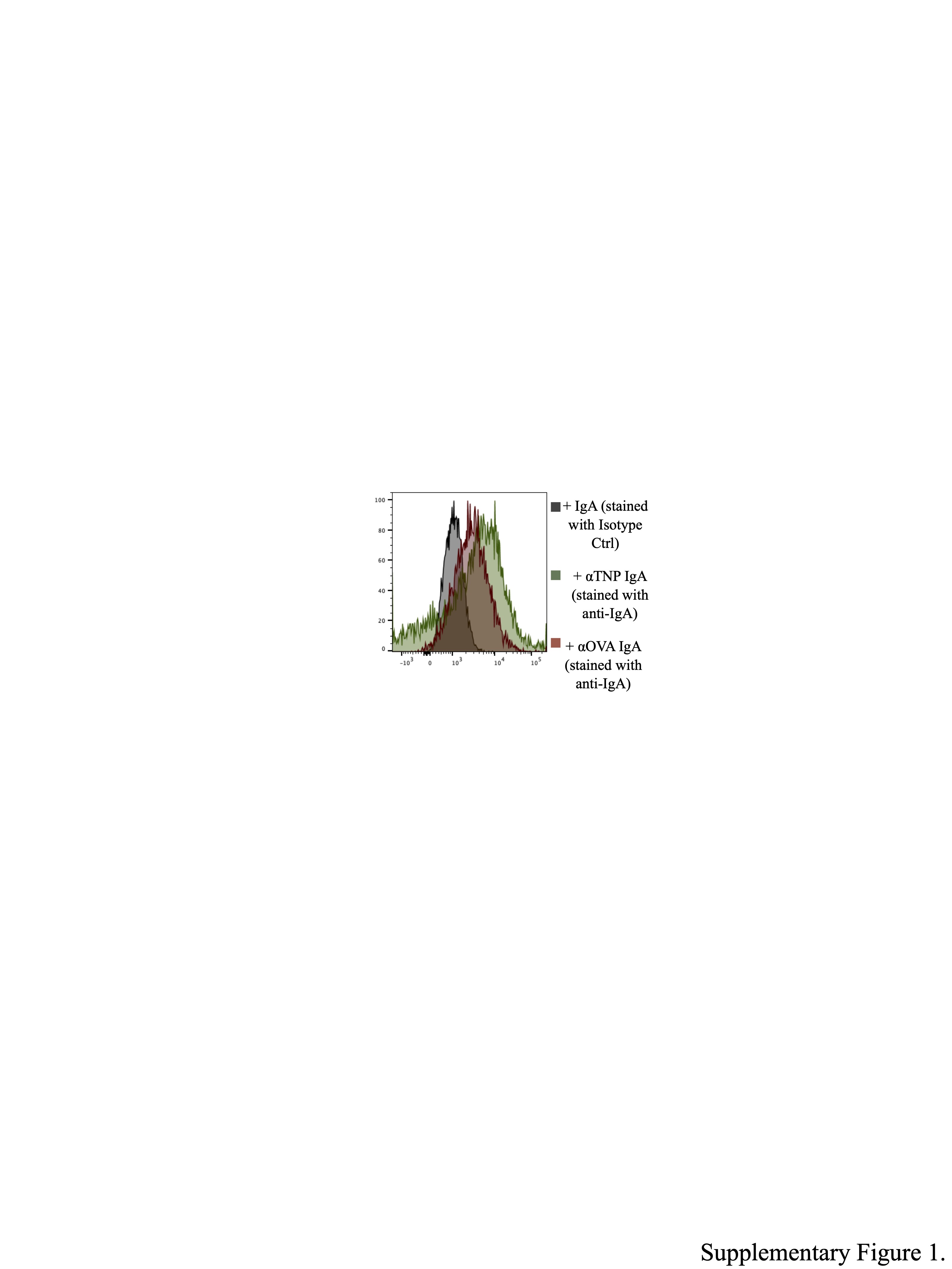

Supplement: Supplementary Figure 1 — Surface anti-OVA IgA staining on bone marrow derived mast cells. Representative histogram of αTNP IgA, and αOVA IgA binding to bone marrow derived mast cells (BMMCs). Mast cells incubated with or without anti-TNP IgA or anti-OVA IgA were stained with antibodies for c-Kit, FcϵRIα, and IgA and analyzed by flow cytometry. Data shown representative of one experiment run twice independently. [file Image_1.jpeg]

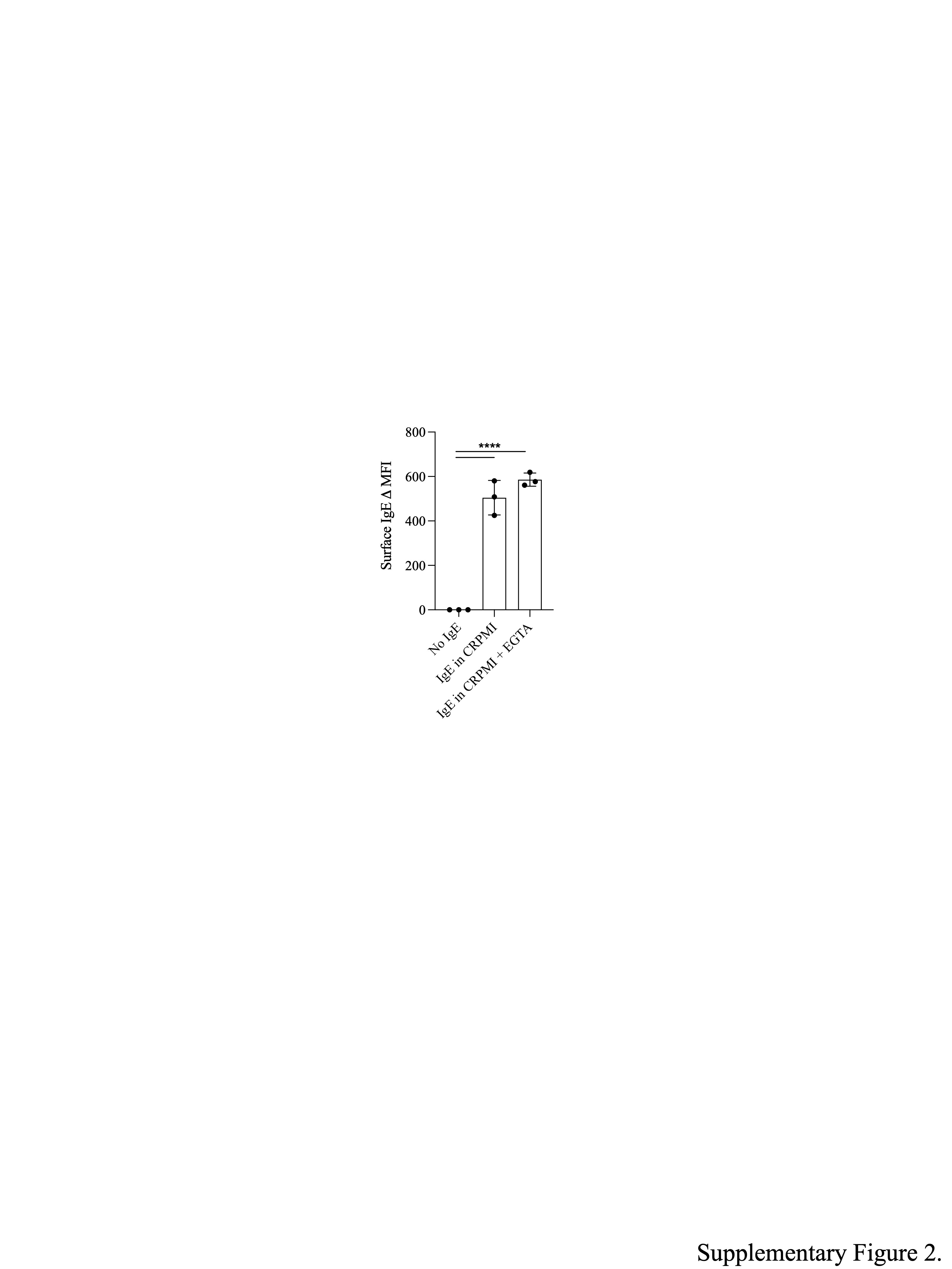

Supplement: Supplementary Figure 2 — Effects of calcium on IgE binding to BMMCs. Mean fluorescence intensity (MFI) of BMMCs stained with anti-IgE after incubation with anti-TNP IgE in the absence or presence of EGTA. Data shown representative of one experiment run twice independently. [file Image_2.jpeg]
